# Quality of Working Life of Nurses and its Related Factors

**Published:** 2014-06-15

**Authors:** Tayebeh Moradi, Farzaneh Maghaminejad, Ismail Azizi-Fini

**Affiliations:** 1 Department of Medical-Surgical Nursing, Faculty of Nursing and Midwifery, Kashan University of Medical Sciences, Kashan, IR Iran; 2 Department of Critical Care Nursing, School of Nursing and Midwifery, Tehran University of Medical Sciences, Tehran, IR Iran

**Keywords:** Quality of Life, Nurses, Job Satisfaction

## Abstract

**Background:**

Nurses as the largest group of health care providers should enjoy a satisfactory quality of working life to be able to provide quality care to their patients. Therefore, attention should be paid to the nurses’ working life.

**Objectives:**

This study aimed to investigate the quality of nurses' working life in Kashans' hospitals during 2012.

**Materials and Methods:**

This cross-sectional study was conducted on 200 nurses during 2012. The data-gathering instrument consisted of two parts. The first part consisted of questions on demographic information and the second part was the Walton’s quality of work life questionnaire. Data were analyzed using the SPSS software. For statistical analysis T test and one way ANOVA were used.

**Results:**

The results of the study showed that 60% of nurses reported that they had moderate level of quality of working life while 37.1% and 2% had undesirable and good quality of working life, respectively. Nurses with associate degrees reported a better quality of working life than others. A significant relationship was found between variables such as education level, work experience, and type of hospital with quality of working life score (P < 0.05). No significant differences were observed between quality of working life score of nurses with employment status (P = 0.061), salary (P = 0.052), age, gender and marital status (P > 0.05).

**Conclusions:**

Nurses' quality of work life was at the moderate level. As quality of work life has an important impact on attracting and retaining employees, it is necessary to pay more attention to the nurses’ quality of work life and its affecting factors.

## 1. Background

Today, quality of work life (QWL) has become an important issue and many studies have been published on this topic (1). This concept was first introduced in the 1930s (2, 3). This concept basically describes the methods by which an organization can ensure the holistic wellbeing of an employee instead of only focusing on work-related aspects. QWL is a process by which the organizations’ employees and stakeholders learn how to work better together to improve both the staff’s quality of life and the organizational effectiveness simultaneously (4, 5). Despite the importance of this issue, an accepted definition for QWL has not yet been introduced (1). Moorhead and Griffin have defined the QWL as the ability of employees to satisfy their important personal needs through what they have learned in their organization (6). In fact, improving the QWL is a comprehensive process to improve the quality of life of employees in the workplace and is essential in any organization to attract and retain its employees (7-10).

The QWL has been studied in various areas, including sociology, psychology, education, management, health care and nursing. In recent decades, QWL has received increasing attention in healthcare settings (11). Health care agencies are one of the largest service providers to the community. Nurses are the largest group of employees in health care organizations (12, 13) and improving their work life quality has became a challenging issue in health care organizations since the 1970s (14, 15). In fact, as a part of the broader quality movement in health care, the QWL concerns of staff development and wellbeing have been recognized as important facets of healthcare organizations’ performance (16). The QWL in health care has been described as strengths and weaknesses in the total work environment (8).

Although nurses have been trained to provide patient care and improve their patients quality of life, but their own needs and their own QWL has been largely ignored (12, 17). Quality of work life is a comprehensive and general schema, which is essential in improving specialized personnel’s satisfaction and attracting and preserving personnel. It also results in positive theories such as increasing profits and provocation (18).

There is an outcry in health services regarding the lack of quality patient care and the poor standard of service delivery. The productivity of nurses is reportedly low. Hall states “to maintain and improve the quality of work life experienced by professional nurses requires that nurses be more skilled and productive in their work settings”. In hospitals where there is a lack of quality of work life, the absenteeism and turnover rates amongst the nurses are usually very high. By assessing and improving the quality of work life, staff performance might increase and burnout among nurses might be reduced. The absenteeism and turnover rates might also decrease (18).

Studies have shown that employees satisfaction of their QWL would not only improve their performance and reduce absenteeism, workplace accidents and job turnover, but also increase their job satisfaction and satisfaction of other aspects of life (2, 4, 7, 10, 19-23). Studies show that satisfied employees work with greater interest, are more loyal to the organization and increase productivity (11, 24). 

However, a number of studies have reported that the quality of nurses work life is seriously impaired (6). Studies have shown that nurses have an average QWL (5, 7, 12, 15, 19, 24). A number of studies have also been conducted on this issue in Iran. In a study, Sharhraky Vahed et al. reported that 65.5% of staff had a relatively desirable QWL (9). Nayeri et al. reported that only 3.6% of nurses were satisfied with their work (7). However, in a study by Dargahi et al. it was reported that most nurses are dissatisfied with most aspects of their QWL and feel that they have a poor work life (10). 

The nurses’ dissatisfaction with their own work life can cause problems such as job dissatisfaction, emotional exhaustion, burn out and job turnover. These factors would in turn affect the quality of care provided by nurses (4, 10, 12, 19). The organization’s success in achieving its goal depends on the quality of human resources. Therefore, attention should be paid to the nurses’ physical and emotional needs (14).

## 2. Objectives

Considering previous studies that reported nurses dissatisfaction of their working conditions in most hospitals in Iran, and the lack of studies in Kashan, this study was conducted to evaluate the quality of working life and its affecting factors of nurses in educational hospitals of Kashan University of Medical Sciences. The results of this study may be an effective step towards improving the quality of nurses working life.

## 3. Materials and Methods

This cross-sectional study was conducted on 200 nurses in hospitals of Kashan during 2012. Sample size was calculated based on a previous study in which Goudarznand-Chegini et al. studied the quality of work life of the employees in public hospitals in Rasht, according to whom the quality of work-life was 73.28 ± 15.26 (2). On this basis, 150 patients were estimated to be needed in this study based on the following parameters α = 0.05, S1 = 15.26, S2 = 8.64, d = 3 (2). However, 200 nurses were selected in this study based on the recommendation of the review board, to compensate possible attrition.

Nurses with diplomas, associate and bachelor's degrees or higher who were working in a hospital and were willing to participate in the study were recruited. 

The samples were selected through quota sampling, and based on the numbers of nursing staff in each hospital the required samples were randomly selected from the list of nurses working at each hospital. Thus, 80% of nurses from general hospitals, 10% of nurses from ear, nasal and throat (ENT) specialty hospitals and 10% of nurses from psychiatric care hospitals were recruited in the study. 

After selecting the participants, the researcher referred to them during their working shifts, invited them to take part in the study and explained the study aims and if they agreed to take part, the questionnaire was given to them individually and they were requested to respond and return it back to the researcher within one day. All nurses completed and returned the questionnaire. 

The data-gathering instrument consisted of two parts. The first part consisted of questions on demographic information (including gender, age, education, marital status, type of hospital, monthly salary). The second part was the Walton’s quality of work life questionnaire. The questionnaire included 35 five choice answers from completely dissatisfied (= 1) to completely satisfied (= 5). 

The QWL questionnaire evaluated the quality of 8 domains of work life including 'adequate and fair compensation', ' work and total life space', 'opportunity for continuous growth and job security', 'opportunity to develop human capacities', ' safe and healthy working environment', ' flexible work schedule and job assignment', ' attention to job design' and ' employee relations' (11). The minimum possible score was 35 and the maximum score was 175. A score from 35 to 80 was considered as poor QWL and scores ranging from 81-130 and 130-175 were considered as moderate and good QWL, respectively (11). 

The content validity of the tool was confirmed by 10 faculty members in KAUMS. Khaghanizadeh et al. in their research on the relationship between occupational stress and the quality of nurses’ work life in selected hospitals of armed forces used a nominal method to evaluate the justifiability of the Iranian questionnaire of quality of work life. Furthermore another method was used to determine the questionnaires perpetuity and its correlation coefficient was calculated as 0.9, which showed a desirable correlation coefficient for the questions. A primary study to determine the justifiability and perpetuity of the questionnaire was performed, which resulted in a Cronbach's alpha of 0.95 (15). Also the questionnaire’s reliability has been examined by several previous studies and the reliability coefficient was reported to be between 0.86 and 0.95 (7, 10, 24).

### 3.1. Ethical Considerations 

The ethical aspect of this study was approved by the institutional ethics committee. Permissions were also obtained from the authorities of the university and hospital officials before data collection. All participants signed a written informed consent in which the purposes of the study were explained and they were assured of the confidentiality of their personal information. 

### 3.2. Statistical analysis 

Data were analyzed using the statistical package for social sciences (SPSS) software. Descriptive statistics were calculated and independent sample t-test was used to examine the relationship of quality of work life and marital status, and gender. Also one-way ANOVA was used to determine the relationship between quality of work life and other demographic variables. P values less than 0.05 were considered significant for all tests. 

## 4. Results

In total, 200 nurses working in Kashan’s hospitals participated in this study. All nurses completed and returned the questionnaire. Amongst all participants 49.7% (n = 99) were between the ages of 30-40 years, 81.4% (n = 162) were female and 79.4% were married. Also, 88.5% (n = 177) had a bachelor's degree and 46.5% had 5 to 10 years of nursing experience. In total, 93% of the participants (n = 186) had a monthly salary between 500.000 to one million Tomans (200-400 dollars). Also, 81% (162 people) worked in a general hospital, 9% (18 people) in an ear nose and throat hospital, and 10% (n = 20) in a psychiatric care hospital. The mean score of overall quality of work life for nurses was 84.36 ± 21.64. Regarding the QWL, 60% (n = 92) of nurses reported that they had a moderate level of work life quality while 37.1% (n = 56) and 2% (n = 3) had undesirable and good level, respectively. Nurses with associate degrees reported a better QWL than others. A significant relationship was found between education level and QWL score (P = 0.04). Post-hoc tests showed that there were significant differences between the QWL of nurses with associate degrees and those with a nursing diploma (P = 0.04) or a master degree (P = 0.05) (Table 1) . 

Nurses with professional experience of more than 15 years had a better QWL than others. A significant correlation was observed between work experience and QWL score (P = 0.01). Tukey pos-hoc test showed that a significant difference existed between the QWL score of nurses with work experience of 5-10 years and those with more than 15 years of work experience (P= 0.01) (Table 1). 

Also, nurses in Ear Nose and Throat specialty hospital reported a better QWL than others. A significant difference was observed between QWL score of nurses in different hospitals (P = 0.03) (Table 1) .

One-way ANOVA test was used and no significant differences were observed between the QWL score of nurses with different employment status (P = 0.061), salary (P = 0.052) and ages (P > 0.05) (Table 1). Also, T-test was used and no significant differences were observed between the QWL scores of nurses with different genders or marital status (P > 0.05) (Table 2). 

**Table 1:** Quality of Work Life by Demographic Variables Using One Way ANOVA

	Mean ± SD	F Value	P Value
**Education**		2.71	0.04
Diploma or Less	75 ± 17.26		
Associate Degree	105 ± 17.02		
Bachelor	83.86 ± 21.40		
Master Degree	80 ± 32.35		
**Work Experience, y**		3.43	0.01
< 5	83.22 ± 25.28		
5-10	80.79 ± 19.80		
10-15	88.85 ± 15.79		
> 15	99.57 ± 18.83		
**Type of hospital**		6.00	0.003
General Hospital	81.99 ± 20.81		
Ear Nose and Throat Hospital	103.42 ± 17.86		
Psychiatric Care Hospital	88.12 ± 23.90		
**Employment status**		2.51	0.061
Permanent	93.09 ± 17.45		
Temporary	80.74 ± 21.50		
Contract	88.38 ± 22.99		
Compulsive Governmental Service	82.09 ± 22.10		
**Monthly Salary, Dollars**		3.00	0.052
< 150	64.17 ± 18.77		
150-300	84.92 ± 21.52		
> 300	90 ± 19.77		
**Age, d**		1.23	0.29
20-30	81.19 ± 23.47		
30-40	85.55 ± 20.21		
40-50	91.75 ± 19.53		
> 50	103 ± 0		

**Table 2.** Quality of Work Life by Demographic Variables Using the T-test

Variable	Mean ± SD	T Value	P Value
**Marital Status**		-1.51	0.13
Single	79.06 ± 20.12		
Married	85.64 ± 21.95		
**Gender**		-1.81	0.07
Male	78.22 ± 20.77		
Female	86.03 ± 21.74		

## 5. Discussion

The present study aimed to evaluate QWL and its related factors of nurses working in KAUMS hospitals. The results of the present study showed that the majority of nurses had a moderate level QWL. Studies have shown that nurses have an average QWL (5, 7, 12, 15, 19, 24). In Boonrod's research the overall mean score of the level of quality of working life among professional nurses in Thailand was at a moderate level (11). Dargahi et al. reported that most nurses were not satisfied with all components of their QWL (10). Also Sharhraky Vahed et al. reported that nurses in Isfahan hospitals had poor QWL (9). Nayeri et al. carried out a descriptive study to investigate the relationship between QWL and productivity among 360 clinical nurses working in the hospitals of Tehran University of Medical Sciences. Their findings showed that QWL was at a moderate level among 61.4% of the participants (7). 

It seems that the QWL is influenced by many factors such as salary, personality, occupational accidents, occupational stress, safety regulations and labor discipline, work setting health conditions, welfare facilities and job prospects. Thus, changes in any of these factors may affect the QWL (5, 10, 15). Also Brooks and Anderson, in an assessment of quality of nursing work life in acute care in a Midwestern state, concluded that QWL is influenced by nursing workload. Therefore, the low QWL of nurses in this study may be related to one of the reasons proposed by previous studies (11). Results of the present study showed a significant relationship between nurses QWL and their education level. However, in his study Dargahi et al. couldn't observe a significant relationship between nurses QWL and their education level (10). Also, Sahraki-Vahed et al. reported that there was no significant relationship between the nurses QWL and their education level (9). In this research we found that the QWL of nurses with lower education level was better than nurses with higher education. It seems that nurses with higher education levels have higher expectations of their working life and consequently experience more emotional exhaustion when their work environment does not meet their expectations. Also Lee et al. showed that nurses with higher level of education perceived more occupational stress (26, 27). Thus, nurses with lower level of education will experience a lower level of QWL.

This study showed a significant relationship between QWL and work experience, so that nurses with more work experience had a better QWL. This finding is consistent with the results of the study by Dargahi et al. (10). Sahrakivahed et al. also reported that employees with more than 20 years of experience had a better QWL than those with less work experience (9). However, Nayeri et al. and Boonrod reported that they could not observe a significant relationship between QWL and the length of work experience (7, 19). One of the sources of occupational stress for nurses is shorter length of work experience (25). Thus, it seems that employees with greater work experience feel less occupational stress and more stability in their job and thus experience a better QWL (9).

The current study revealed a significant relationship between nurses QWL and the type of hospital so that nurse in specialty settings such as ENT hospitals had a better QWL than nurses in general hospitals. The differences in QWL of nurses in various hospitals could be attributed to the hospital’s circumstances. It has been reported that factors such as hospital size, number and type of patients, nurse’s salary, hospital policies and physical environment may affect the nurses QWL. Dargahi et al. also reported that nurses in small size hospitals had greater satisfaction with their QWL (10). In addition, Nabirye's research showed that nurses in public hospitals reported higher levels of occupational stress and lower levels of job satisfaction and performance (7). Thus, the better QWL of nurses in ENT hospitals can be attributed to their specialty work setting, higher salary and lower level of stress (10, 11).

The present study showed that nurses with permanent employment had a better QWL than nurses with other employment statuses. This finding is consistent with the results of Sharhraky Vahed et al. (9). It seems that higher income and better career prospects and job stability of nurses with permanent employment results better QWL compared to nurses with temporary or contract employment (10). In the present study, although female nurses had a higher QWL mean score than male nurses, the difference was not significant. This finding is consistent with the results of Nayeri et al. and Darghahi et al. and incongruent with the study by Heydari-Rafat et al. who reported that female nurses had a better QWL than male nurses (5). In contrast, male nurses had a better QWL in a study conducted by Sharhraky Vahed et al. (9). However, the lower mean QWL of male nurses may be due to the fact that male nurses usually participate in more stressful nursing activities and this may negatively affect their perceived QWL (15).

The current study could not reveal a significant relationship between QWL and marital status. Two other studies have also shown that QWL has no significant relationship with marital status (7, 10). However, Khaghanizadeh et al. reported that 82% of married and 66% of single individuals had a moderate level of QWL (15). In this study, the QWL was higher in married nurses than single individuals although the difference was not statistically significant. This could perhaps be because married nurses receive greater emotional support from their spouses and this decreases their stresses and thus, they experience a better QWL and job satisfaction (9).

The results of the present study showed that there was no significant correlation between age and QWL. Two other studies also reported that there was no significant relationship between age and QWL (10, 19). These findings are not consistent with the report by Dehghan Nayeri et al., suggesting that there is a close correlation between age and QWL (7). On the other hand, Khaghani et al. reported that there is an inverse correlation between age and QWL (15). The present study showed that nurses' quality of work life is at the moderate level. As QWL has an important impact on attracting and retaining employees, it is necessary to pay more attention to the nurses QWL and its affecting factors. The authorities in the health care system should develop strategies for improving the nurses work conditions and their QWL, so that, nurses will be able to perform better care for their patients. This research provides an initial step in understanding the work life of nurses in an Iranian setting. Also, there is a need for outcome-driven research examining the effectiveness, efficacy and cost-benefits of specific strategies aimed at improving the QWL of nurses. When using the results of this study it should be noted that we used a self-report instrument and this may affect the results. Thus, further studies should be conducted with more objective instruments.
